# Modified staging system of positive lymph nodes based nomogram in intrahepatic cholangiocarcinoma

**DOI:** 10.1186/s12935-023-03005-6

**Published:** 2023-07-29

**Authors:** Chongyu Zhao, Xiyuan Li, Li Luo, Cheng Chen, Chaobin He

**Affiliations:** 1 Department of Hepatobiliary Surgery, Xinqiao Hospital, Army Medical University, Chongqing, P. R. China; 2 State Key Laboratory of Oncology in South China, Collaborative Innovation Center for Cancer Medicine, Sun Yat-sen University Cancer Center, Guangzhou, Guangdong, 510060 P. R. China; 3 Department of Head and Neck Surgery, Sun Yat-sen University Cancer Center, Guangzhou, Guangdong, 510060 P. R. China; 4 Department of Cardiology, The First Affiliated Hospital of Dalian Medical University, Dalian, P. R. China; 5 Department of Pancreatobiliary Surgery, Sun Yat-sen University Cancer Center, Guangzhou, Guangdong, 510060 P. R. China

**Keywords:** Intrahepatic cholangiocarcinoma, Lymph nodes metastasis, Lymph node staging system

## Abstract

**Background:**

Intrahepatic cholangiocarcinoma (iCCA) presents the similar trend and prevalence of lymph node metastasis to other biliary tract cancer. There is still a necessity and possibility for the current classification of lymph node in the 8th TNM of iCCA, which is the same as the criteria of hepatoma carcinoma (HCC), to further improve the prognostic capacity. We aim to explore the optimal positive lymph nodes cutoff value that could predict the survival outcomes of patients with iCCA and further establish a prognostic nomogram.

**Method:**

Clinical characteristics were retrospectively collected in 292 patients with iCCA from Sun Yat-sen University Cancer Center (SYSUCC) for preliminary analysis. A retrospective analysis of 107 patients with iCCA in the First Hospital of Dalian Medical University (FHDMU) was performed for verification. R software was used to determine the optimal cutoff value of positive lymph nodes (PLN) and further establish the nomogram with the Cox regression model in the primary cohort.

**Results:**

In those patients who were graded into the N1 stage in 8th TNM staging system, the patients with PLN between 1 and 3 showed significantly better overall survival than those patients with more than 4 PLN (P < 0.0001). Moreover, there was a significant correlation between the new PLN classification and adverse clinical characteristic including Micro Invasion (P = 0.001), Lymph Vessel Invasion (P = 0.040), Satellite Sites (P < 0.001), and Tumor Size (P = 0.005). The PLN and ELN were both independent prognostic factors for survival outcomes in the multivariate analysis, and further showed large contribution to the nomogram. The nomogram achieved a satisfied C-index of 0.813 for overall survival (OS), 0.869 for progression-free survival (PFS) in the primary cohort, and 0.787 for OS, 0.762 for PFS in the validation cohort.

**Conclusion:**

The modified classification of PLN in iCCA could accurately stratify the N1 stage patients in 8th TNM staging system into two groups with significantly different overall survival. The development of this nomogram can offer new evidence to precisely post-operative management of iCCA patients.

**Supplementary Information:**

The online version contains supplementary material available at 10.1186/s12935-023-03005-6.

## Introduction

Intrahepatic cholangiocarcinoma (iCCA) consists of malignant tumorous cells with heterogeneous natures, with origination from the biliary tract, hence the pathological characteristics of the biliary tract [1], or trans-differentiation from hepatocytes [2, 3]. Radical surgical resection is the only available treatment option that improves long-term survival for iCCA patients [4]. It has been well established that the overall survival of iCCA patients ranges from 17 to 42% after surgery [5, 6].

Lymph node (Ln) dissection has been regularly conducted in surgery of iCCA for many years [1, 4]. The 8th edition of TNM stage system recommends the dissection of at least 6 lymph nodes in iCCA for an accurate N staging and further defined nodal involvement only as present or absent [7]. The recommendation and definition were similar to those of hepatoma carcinoma (HCC) [8]. Indeed, there were objective similarities in anatomical location between iCCA and HCC. However, the origination of these two malignancies was totally different. Moreover, iCCA followed the pattern of gradual invasion from the primary site to local lymph nodes before metastasis which was a resemblance to other biliary tract cancer (BTC) [9]. Otherwise, intrahepatic metastasis tended to occur previously in HCC. In view of the above, the N stage of iCCA was worthy to reconsider.

Besides the number of positive LNs (PLN), there were plenty of studies illustrate that examined lymph node (ELN) count and lymph node ratio (LNR) were significant prognostic factors for various cancer, such as penile cancer [10], pancreatic cancer [11], and gallbladder cancer [12]. Insufficient ELN may lead to mis-staging of the N category [12]. Rather than a simple binary designation of LN status, LNR, which was defined as the ratio of the number of positive LNs relative to the ELN, has been proposed to be a sensitive indicator of survival outcome in various malignancies.

Therefore, this present study aimed to compare those lymph nodes related indexes and demonstrate the optimal cutoff value of the most powerful indicator in iCCA patients. Then further establishing a prognostic nomogram to predict the survival outcomes in patients with iCCA after surgical resection based on multicenter cohorts.

## Method

### Patients’ characteristics

A total of 396 patients diagnosed with iCCA through pathological examination and underwent radical surgical resection at Sun Yat-sen University Cancer Center (SYSUCC) or the first affiliated hospital of Dalian Medical University (FHDMU) were enrolled in the current study. The 289 patients from SYSUCC between January 2000 and December 2018 served as the primary cohort while 107 patients from FHDMU between May 2013 and December 2019 served as the validation cohort. Preoperative baseline characteristics, liver function, tumor markers, pathological examinations, tumor progression, and time to death or last visit were collected and displayed in Table 1. The indications and contraindications to resection surgeries were the same for both cohorts of this study. Written informed consents were obtained from all patients enrolled in this study. The design and execution of the study were approved by the ethics committees of both participating centers.

**Table 1:** Clinical and pathological characteristics of the SYSUCC cohort and FHDMU cohort

Variables	Primary cohort (n = 289)	Validation cohort (n = 107)	Variables	Primary cohort (n = 289)	Validation cohort (n = 107)
Gender			Macrovascular invasion		
Male	178 (61.6%)	62 (57.9%)	Absence	271 (93.8%)	95 (88.8%)
Female	111 (38.4%)	45 (42.1%)	Presence	18 (6.23%)	12 (11.2%)
Age (years)			Satellite sites		
≤ 60 years	189 (65.4%)	33 (30.8%)	Absence	198 (68.5%)	106 (99.1%)
> 60 years	100 (34.6%)	74 (69.2%)	Presence	91 (31.5%)	1 (0.93%)
WBC count (×10^9^/L)			Adjacent Organ Invasion		
≤ 10	256 (88.6%)	92 (86.0%)	Absence	257 (88.9%)	103 (96.3%)
> 10	33 (11.4%)	15 (14.0%)	Presence	32 (11.1%)	4 (3.74%)
HGB (g/L)			Tumor size		
≤ 175	125 (43.3%)	30 (28.0%)	≤ 5 cm	112 (38.8%)	52 (48.6%)
> 175	164 (56.7%)	77 (72.0%)	≤ 5 cm	177 (61.2%)	55 (51.4%)
PLT (×109/L)			LN metastasis		
≤ 350	10 (3.46%)	5 (4.67%)	Absence	247 (85.5%)	95 (88.8%)
> 350	279 (96.5%)	102 (95.3%)	Presence	42 (14.5%)	12 (11.2%)
ALT (U/L)			Positive LN number:		
≤ 50	251 (86.9%)	55 (51.4%)	0	247 (85.5%)	95 (88.8%)
> 50	38 (13.1%)	52 (48.6%)	1	18 (6.23%)	3 (2.80%)
AST (U/L)			2	10 (3.46%)	4 (3.74%)
≤ 40	251 (86.9%)	56 (52.3%)	4	6 (2.08%)	2 (1.87%)
> 40	38 (13.1%)	51 (47.7%)	5	4 (1.38%)	2 (1.87%)
ALP (U/L)			6	3 (1.04%)	-
≤ 125	179 (61.9%)	25 (23.4%)	9	-	1 (0.93%)
> 125	110 (38.1%)	82 (76.6%)	12	1 (0.35%)	-
GGT (U/L)			Tumor differentiation		
≤ 60	106 (36.7%)	16 (15.0%)	Low	32 (11.1%)	13 (12.2%)
> 60	183 (63.3%)	91 (85.0%)	Medium/High	257 (88.9%)	94 (87.8%)
ALB (g/L)			T stage 8th		
> 40	4 (1.4%)	38 (35.5%)	1	68 (23.5%)	84 (78.5%)
≤ 40	285 (98.7%)	69 (64.5%)	2	44 (15.2%)	5 (4.67%)
TBIL (µmol/L)			3	153 (52.9%)	14 (13.1%)
≤ 20.5	262 (90.7%)	54 (50.5%)	4	24 (8.30%)	4 (3.74%)
> 20.5	27 (9.34%)	53 (49.5%)	N stage 8th		
IBIL (µmol/L)			Absence	247 (85.5%)	89(83.1%)
≤ 15	272 (94.1%)	65 (60.7%)	Presence	42 (14.5%)	18(16.9%)
> 15	17 (5.88%)	42 (39.3%)	TNM 8th		
HBsAg			IA	31 (10.7%)	35 (32.7%)
Absence	160 (55.4%)	-	IB	36 (12.5%)	46 (43.0%)
Presence	129 (44.6%)	-	II	37 (12.9%)	2 (1.87%)
CA19-9 (U/ml)			IIIA	125 (43.3%)	8 (7.48%)
≤ 35	140 (48.4%)	25 (23.4%)	IIIB	60 (20.8%)	16 (15.0%)
>35	149 (51.6%)	82 (76.6%)	After operation therapy		
CEA (ng/ml)			Absence	159 (55.0%)	72 (67.3%)
≤ 5	208 (72.0%)	60 (56.1%)	Presence	130 (45.0%)	35 (32.7%)
> 5	81 (28.0%)	47 (43.9%)	LN5 metastasis		
NLR			Absence	288 (99.7%)	
< 2.62	191 (66.1%)	36 (33.6%)	Presence	1 (0.35%)	
≥ 2.62	98 (33.9%)	71 (66.4%)	LN7 metastasis		
PLR			Absence	284 (98.3%)	106 (99.1%)
< 104.85	169 (58.5%)	24 (22.4%)	Presence	5 (1.73%)	1 (0.93%)
≥ 104.85	120 (41.5%)	83 (77.6%)	LN8 metastasis		
SII			Absence	280 (96.9%)	101 (94.4%)
0	66 (22.8%)	30 (28.0%)	Presence	9 (3.10%)	6 (5.60%)
1	223 (77.2%)	77 (72.0%)	LN9 metastasis		
LCR			Absence	283 (97.9%)	-
0	21 (7.27%)	-	Presence	6 (2.08%)	-
1	268 (92.7%)	-	LN12 metastasis		
PNI			Absence	262 (90.7%)	96 (89.7%)
0	274 (94.8%)	48 (44.9%)	1	23 (7.96%)	8 (7.48%)
1	15 (5.19%)	59 (55.1%)	2	3 (1.04%)	2 (1.87%)
PI			4	1 (0.35%)	-
0	217 (75.1%)	32 (29.9%)	5	-	1 (0.93%)
1	61 (21.1%)	63 (58.9%)	LN13 metastasis		
2	11 (3.81%)	12 (11.2%)	Absence	281 (97.3%)	102 (95.3%)
mGPS			Presence	8 (2.7%)	5 (4.7%)
0	230 (79.6%)	35 (32.7%)	LN14 metastasis		
1	56 (19.4%)	42 (39.3%)	Absence	288 (99.7%)	-
2	3 (1.04%)	30 (28.0%)	Presence	1 (0.35%)	-
Microvascular invasion			LN16 metastasis		
Absence	234 (81.0%)	86 (89.7%)	Absence	286 (99.0%)	-
Presence	55 (19.0%)	11 (10.3%)	Presence	3 (1.04%)	-
Lymph-vessel invasion					
Absence	270 (93.4%)	-			
Presence	19 (6.57%)	-			

### Follow-up and survival outcomes

30 days post successful resection, routine post-operative follow-up began with a frequency of every three months for the first year and every six months until death or drop-out of the study. During routine follow-up, the patterns and timing of recurrence were determined regular abdominal CT, carcinoembryonic antigen (CEA) measurement, and carbohydrate antigen 19 − 9 (CA19-9) measurement. Additional imaging examinations were performed as necessary to assist in determining patterns of recurrence. Medical records of the two cohorts were retrieved on November 30, 2020.

### Statistical analysis

Data collected from the medical records were analyzed in whole numbers and proportions. Proportions were compared using the chi-square test or the Fisher Exact test. Mann-Whitney U test was utilized to compare the distributions of continuous variables. Survival curves were generated using the Kaplan-Meier method and then the log-rank test was performed to compare between groups. The multivariate analysis of the predictive factors of PPS was conducted using the Cox regression model. Then the nomogram was generated using the multivariate analysis in the training cohort. The evaluation of the predictive performance calculated with Harrell’s concordance index (C-index) was carried out with both the calibration curves and survival curves. All statistical analyses were performed using SPSS software version 22 (SPSS inc., Chicago, IL, USA) or R software version 4.1.1 (R Development Core Team; http://www.r-project.org). All statistical analyses were on the basis of two-sided p values. p-values < 0.05 are considered as statistical significance.

## Results

### Characteristics of patients

The demographics, pre-operative clinical diagnostics, surgical and post-operative pathological characteristics of the recurred iCCA patients in both cohorts were displayed in Table 1. 38.4% of the patients in the primary cohort were females while 42.1% of the validation cohort were females. The median age was 56 years of age and 64 years of age, for the primary cohort or validation cohort, respectively. Further, 32.7% of the patients were prescribed chemotherapy after resection surgeries. No significant difference in baseline clinical characteristics was observed between the two cohorts.

The general survival outcomes were shown as followed: in the primary cohort, the 1-, 2-, 3-year OS rates were 78.2%, 64.9%, and 52.2%, respectively; the 1-, 2-, 3-year PFS were 48.5%, 35.4%, and 31.5% while the 1-, 2-, 3-year PPS were 49.6%, 30.5%, and 19.8%. On the other hand, in the validation cohort, the 1-, 2-, 3-year OS rates were 61.8%, 40.4%, and 32.7%; the 1-, 2-, 3-year PFS were 44.7%, 29.3%, and 21.0%; the 1-, 2-, 3-year PPS were 53.8%, 24.3%, and 2.6%, respectively.

### Modified classification of PLN in iCCA

**Fig. 1:**
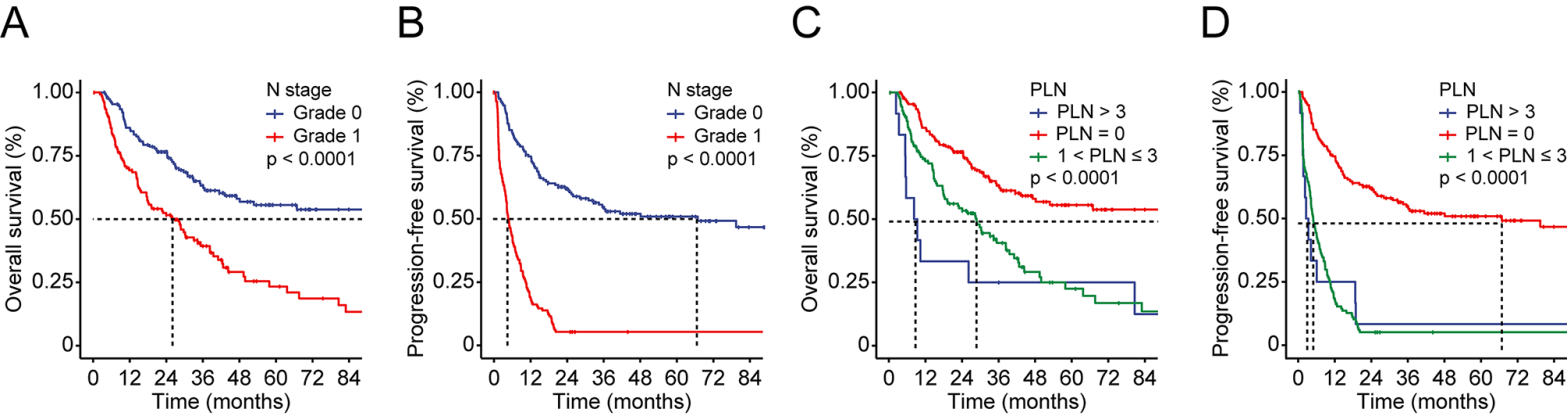
**Kaplan-Meier curves for OS and PFS in patients with iCCA stratified by the N stage of 8th TNM staging system and modified PLN staging system**. (**A**) OS curve stratified by N stage of 8th TNM staging system; (**B**) PFS curve stratified by N stage of 8th TNM staging system; (**C**) OS curve stratified by modified PLN staging system; (**D**) PFS curve stratified by modified PLN staging system

The overall survival (OS) curves and progression-free survival (PFS) curves were conducted according to the existing N stage classification of the 8th TNM stage in iCCA and other BTC in order to compare their stratified ability of survival outcomes in iCCA. As presented in Fig. 1, the new cut-off value of 3 in PLN could further stratify those patients who were graded in N stage 1 according to the 8th TNM stage system in iCCA. Those patients with more than 4 PLN manifested poorer overall survival than the patients with PLN numbers between 1 and 3.

### Clinical characteristics stratified by new PLN classification

**Fig. 2:**
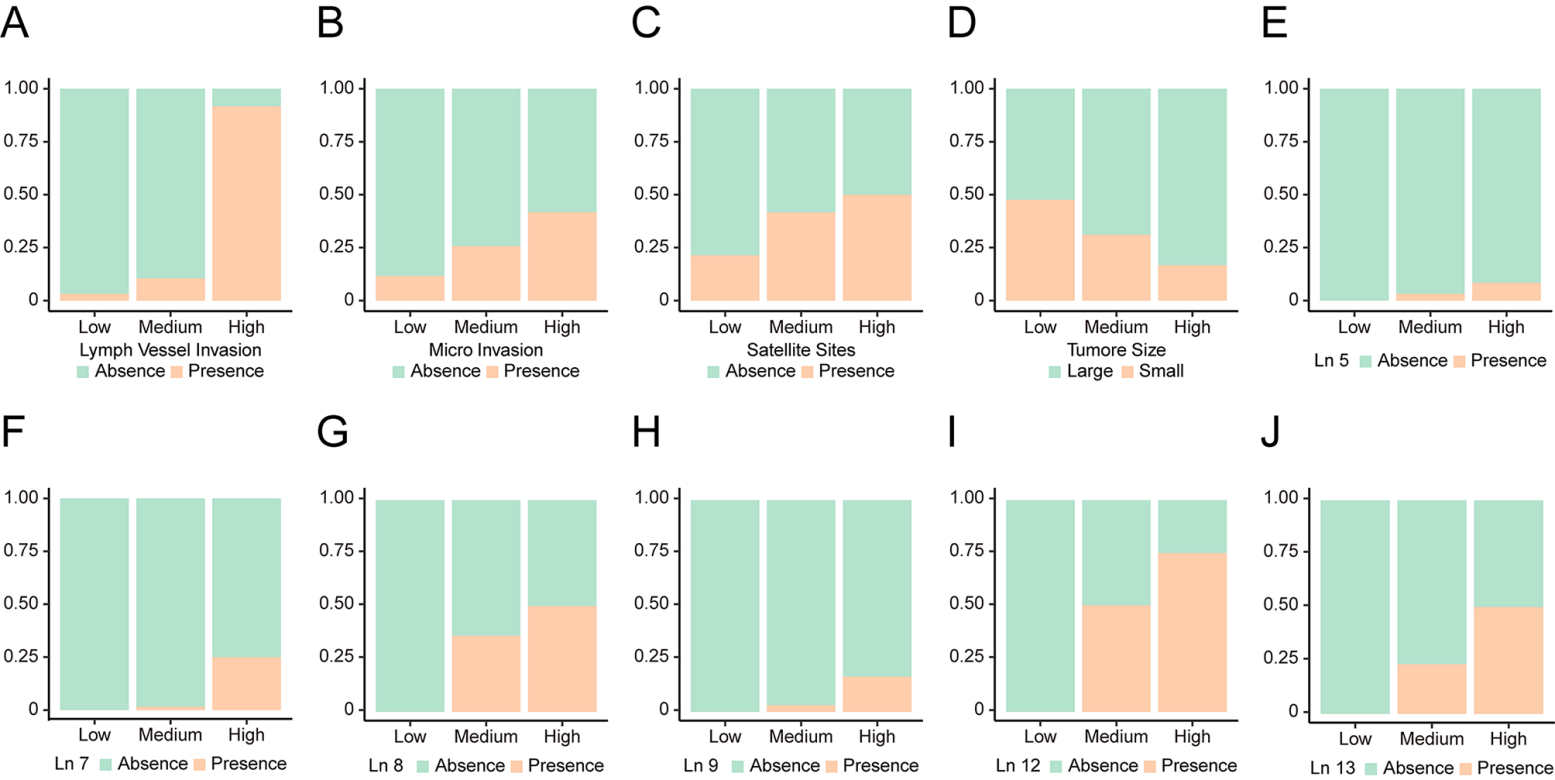
**Comparison of clinical characteristics of iCCA patients with different modified PLN staging system**. (**A**) Lymph vessel invasion; (**B**) Micro invasion; (**C**) Satellite sites; (**D**) Tumor size; E, Ln 5; F, Ln 7; G, Ln 8; H, Ln 9; I, Ln 12; J, Ln 13

With the new PLN classification, those iCCA patients were divided into 3 groups: Low (n = 155, 53.08%), Medium (n = 125, 42.81%), High (n = 12, 4.11%). As shown in Table 2, the new positive lymph nodes classification in iCCA were remarkably correlated with 17 factors: OS months (P = 0.001), PFS months (P < 0.001), Prognostic Index (PI) (P = 0.005), modified Glasgow Prognostic Score (mGPS) (P = 0.001), Micro Invasion (P = 0.001), Lymph Vessel Invasion (P = 0.040), Satellite Sites (P < 0.001), Tumor Size (P = 0.005), N stage of 8th TNM (P = 0.049), After Operative Therapy (P < 0.001), Ln 5 (P = 0.021), Ln 7 (P < 0.001), Ln 8 (P < 0.001), Ln 9 (P = 0.002), Ln 12 (P < 0.001), Ln 13 (P < 0.001), and Ln 16 (P < 0.001). More detailed distribution of these significant factors stratified by the new PLN classification were demonstrated in Fig. 2.

**Table 2:** clinical characteristic stratified by Positive LN number (PLN)

Characteristics	ALL	High	Medium	Low	P value	Characteristics	ALL	High	Medium	Low	P value
	N = 292	N = 12	N = 125	N = 155			N = 292	N = 12	N = 125	N = 155	
OS months	34.7 (32.3)	31.2 (52.7)	26.8 (27.2)	41.4 (32.8)	0.001	Liver Capsule Invasion					0.154
PFS months	24.5 (32.9)	20.4 (51.5)	8.79 (19.4)	37.5 (34.4)	<0.001	Absence	114 (39.0%)	6 (50.0%)	41 (32.8%)	67 (43.2%)	
Progression Period					0.093	Presence	178 (61.0%)	6 (50.0%)	84 (67.2%)	88 (56.8%)	
Early	192 (65.7%)	10 (83.3%)	96 (76.8%)	86 (55.5%)		Tumor Differetiation					0.574
Late	100 (34.3%)	2 (16.7%)	29 (23.2%)	69 (44.5%)		High	185 (63.4%)	9 (75.0%)	82 (65.6%)	94 (60.6%)	
Gender					0.628	Low	6 (2.05%)	0 (0.00%)	1 (0.80%)	5 (3.23%)	
Female	111 (38.0%)	5 (41.7%)	51 (40.8%)	55 (35.5%)		Medium	101 (34.6%)	3 (25.0%)	42 (33.6%)	56 (36.1%)	
Male	181 (62.0%)	7 (58.3%)	74 (59.2%)	100 (64.5%)		T					.
Age					0.825	Grade 1	34 (11.6%)	0 (0.00%)	12 (9.60%)	22 (14.2%)	
>60	103 (35.3%)	3 (25.0%)	45 (36.0%)	55 (35.5%)		Grade 2	37 (12.7%)	3 (25.0%)	8 (6.40%)	26 (16.8%)	
≤60	189 (64.7%)	9 (75.0%)	80 (64.0%)	100 (64.5%)		Grade 3	44 (15.1%)	3 (25.0%)	21 (16.8%)	20 (12.9%)	
WBC					0.168	Grade 4	153 (52.4%)	6 (50.0%)	71 (56.8%)	76 (49.0%)	
Elevated	33 (11.3%)	2 (16.7%)	18 (14.4%)	13 (8.39%)		Grade 5	24 (8.22%)	0 (0.00%)	13 (10.4%)	11 (7.10%)	
Normal	259 (88.7%)	10 (83.3%)	107 (85.6%)	142 (91.6%)		N					0.049
ALB					0.086	Grade 0	155 (53.1%)	7 (58.3%)	56 (44.8%)	92 (59.4%)	
Decreased	5 (1.71%)	1 (8.33%)	3 (2.40%)	1 (0.65%)		Grade 1	137 (46.9%)	5 (41.7%)	69 (55.2%)	63 (40.6%)	
Normal	287 (98.3%)	11 (91.7%)	122 (97.6%)	154 (99.4%)		TNM					.
TBIL					0.555	IA	34 (11.6%)	0 (0.00%)	12 (9.60%)	22 (14.2%)	
Elevated	27 (9.25%)	2 (16.7%)	11 (8.80%)	14 (9.03%)		IB	36 (12.3%)	3 (25.0%)	7 (5.60%)	26 (16.8%)	
Normal	265 (90.8%)	10 (83.3%)	114 (91.2%)	141 (91.0%)		IIA	36 (12.3%)	0 (0.00%)	15 (12.0%)	22 (14.15%)	
IBIL					0.106	IIIA	125 (42.8%)	1 (8.33%)	51 (40.8%)	73 (47.1%)	
Elevated	17 (5.82%)	2 (16.7%)	9 (7.20%)	6 (3.87%)		IIIB	60 (20.5%)	8 (66.7%)	40 (32.0%)	12 (7.74%)	
Normal	275 (94.2%)	10 (83.3%)	116 (92.8%)	149 (96.1%)		After Operation Therapy					<0.001
NLR					0.466	Absence	161 (55.1%)	4 (33.3%)	44 (35.2%)	113 (72.9%)	
Grade 0	194 (66.4%)	6 (50.0%)	83 (66.4%)	105 (67.7%)		Presence	131 (44.9%)	8 (66.7%)	81 (64.8%)	42 (27.1%)	
Grade 1	98 (33.6%)	6 (50.0%)	42 (33.6%)	50 (32.3%)		LN5					0.021
LMR					0.362	Absence	287 (98.3%)	11 (91.7%)	121 (96.8%)	155 (100%)	
Grade 0	125 (42.8%)	7 (58.3%)	49 (39.2%)	69 (44.5%)		Presence	5 (1.71%)	1 (8.33%)	4 (3.20%)	0 (0.00%)	
Grade 1	167 (57.2%)	5 (41.7%)	76 (60.8%)	86 (55.5%)		LN6					0.469
PI					0.005	Absence	291 (99.7%)	12 (100%)	124 (99.2%)	155 (100%)	
Grade 0	220 (75.3%)	6 (50.0%)	86 (68.8%)	128 (82.6%)		Presence	1 (0.34%)	0 (0.00%)	1 (0.80%)	0 (0.00%)	
Grade 1	61 (20.9%)	4 (33.3%)	34 (27.2%)	23 (14.8%)		LN7					<0.001
Grade 2	11 (3.77%)	2 (16.7%)	5 (4.00%)	4 (2.58%)		Absence	287 (98.3%)	9 (75.0%)	123 (98.4%)	155 (100%)	
mGPS					0.001	Presence	5 (1.71%)	3 (25.0%)	2 (1.60%)	0 (0.00%)	
Grade 0	233 (79.8%)	7 (58.3%)	90 (72.0%)	136 (87.7%)		LN8					<0.001
Grade 1	56 (19.2%)	4 (33.3%)	34 (27.2%)	18 (11.6%)		Absence	241 (82.5%)	6 (50.0%)	80 (64.0%)	155 (100%)	
Grade 2	3 (1.03%)	1 (8.33%)	1 (0.80%)	1 (0.65%)		Presence	51 (17.5%)	6 (50.0%)	45 (36.0%)	0 (0.00%)	
CA199					0.083	LN9					0.002
Elevated	151 (51.7%)	5 (41.7%)	72 (57.6%)	103 (66.4%)		Absence	286 (97.9%)	10 (83.3%)	121 (96.8%)	155 (100%)	
Normal	141 (48.3%)	7 (58.3%)	53 (42.4%)	52 (33.6%)		Presence	6 (2.05%)	2 (16.7%)	4 (3.20%)	0 (0.00%)	
CEA					0.146	LN10					0.469
Elevated	81 (27.7%)	3 (25.0%)	42 (33.6%)	36 (23.2%)		Absence	291 (99.7%)	12 (100%)	124 (99.2%)	155 (100%)	
Normal	211 (72.3%)	9 (75.0%)	83 (66.4%)	119 (76.8%)		Presence	1 (0.34%)	0 (0.00%)	1 (0.80%)	0 (0.00%)	
Micro Invasion:					0.001	LN12					<0.001
Absence	237 (81.2%)	7 (58.3%)	93 (74.4%)	137 (88.4%)		Absence	220 (75.3%)	3 (25.0%)	62 (49.6%)	155 (100%)	
Presence	55 (18.8%)	5 (41.7%)	32 (25.6%)	18 (11.6%)		Presence	72 (24.7%)	9 (75.0%)	63 (50.4%)	0 (0.00%)	
Lymph Vessel Invasion:					0.040	LN13					<0.001
Absence	263 (90.1%)	1 (8.3%)	112 (89.6%)	150 (96.8%)		Absence	257 (88.0%)	6 (50.0%)	96 (76.8%)	155 (100%)	
Presence	29 (9.9%)	11 (91.7%)	13 (10.4%)	5 (3.2%)		Presence	35 (12.0%)	6 (50.0%)	29 (23.2%)	0 (0.00%)	
Satellite Sites					<0.001	LN14					0.469
Absence	201 (68.8%)	6 (50.0%)	73 (58.4%)	122 (78.7%)		Absence	291 (99.7%)	12 (100%)	124 (99.2%)	155 (100%)	
Presence	91 (31.2%)	6 (50.0%)	52 (41.6%)	33 (21.3%)		Presence	1 (0.34%)	0 (0.00%)	1 (0.80%)	0 (0.00%)	
Tumor Size					0.005	LN16					<0.001
>5 cm	177 (60.6%)	10 (83.3%)	86 (68.8%)	81 (52.3%)		Absence	277 (94.9%)	8 (66.7%)	114 (91.2%)	155 (100%)	
≤5 cm	115 (39.4%)	2 (16.7%)	39 (31.2%)	74 (47.7%)		Presence	15 (5.14%)	4 (33.3%)	11 (8.80%)	0 (0.00%)	

### Prognostic factors of overall survival and progression-free survival

22 clinical factors, which included hematological and pathological elements, were identified as prognostic factors for OS and PFS in univariate analysis (Table 3). Moreover, the Cox-regression analysis was carried out to filtrate the independent prognostic factors of OS and PFS. In the multivariate analysis, only CA19-9 (P = 0.005), CEA (P < 0.001), mGPS (P = 0.036), ELN (P < 0.001), PLN (P = 0.006), and Ln 8 (P = 0.013) displayed statistical difference of OS, and the factors independently associated with PFS were: CA19-9 (P = 0.032), CEA (P = 0.034), ELN (P = 0.041), PLN (P < 0.001), Tumor differentiation (P = 0.002), Tumor size (P = 0.027), and After operation therapy (P = 0.004) (Table 3). Both PLN and ELN were significant prognostic factors in the Cox-regression analysis, to further demonstrate the prognostic predictive capacity of PLN and ELN, the ROC curves and AUROC values were calculated (Supplementary Fig. 1). The performance of PLN in ROC analysis was remarkably more outstanding than that of ELN.

**Table 3:** Univariate and multivariate analysis of prognostic factors of OS and PFS in the SYSUCC cohort

Variables	OS				PFS			
	Univariate		Multivariate		Univariate		Multivariate	
	HR (95% CI)	P	HR (95% CI)	P	HR (95% CI)	P	HR (95% CI)	P
WBC, ×10^9^/L (≤ 10: >10)	1.547 (1.245–1.923)	< 0.001	0.937 (0.444–1.977)	0.864	1.541 (1.019–2.329)	0.040	0.953 (0.635–1.428)	0.814
CA19-9, U/ml (≤ 35: >35)	1.397 (1.184–1.647)	< 0.001	1.796 (1.198–2.692)	0.005	1.939 (1.459–2.575)	< 0.001	1.190 (1.015–1.396)	0.032
CEA, ng/ml (≤ 5: >5)	1.647 (1.393–1.947)	< 0.001	1.525 (1.260–1.846)	< 0.001	1.756 (1.301–2.370)	< 0.001	1.204 (1.014–1.430)	0.034
mGPS		< 0.001		0.036		< 0.001		0.516
0	Ref		Ref		Ref		Ref	
1	0.458 (0.113–1.866)	0.276	1.050 (0.153–7.210)	0.470	0.503 (0.160–1.582)	0.240	0.816 (0.384–1.731)	0.596
2	1.435 (0.346–5.948)	0.619	2.653 (0.434–16.202)	0.035	1.124 (0.350–3.615)	0.8441	1.312 (0.728–2.362)	0.366
NLR (< 2.62:≥2.62)	0.753 (0.639–0.887)	0.001	0.809 (0.529–1.238)	0.329	0.800 (0.694–0.922)	0.002	0.909 (0.752-1.100)	0.329
LMR (< 4.06:≥4.06)	1.203 (1.025–1.413)	0.024	1.078 (0.725–1.602)	0.712	1.152 (1.003–2.324)	0.046	1.040 (0.877–1.233)	0.653
PI		< 0.001		0.469		< 0.001		0.218
0	Ref		Ref		Ref		Ref	
1	0.407 (0.197–0.841)	0.015	2.673 (0.538–13.283)	0.229	0.694 (0.353–1.363)	0.289	1.540 (0.662–3.580)	0.316
2	1.258 (0.590–2.681)	0.552	1.929 (0.643–5.783)	0.241	1.488 (0.731–3.030)	0.273	1.317 (0.906–1.913)	0.149
ELN (Low: High)	0.805 (0.758–0.855)	< 0.001	0.643 (0.579–0.713)	< 0.001	0.565 (0.395–0.808)	0.002	0.808 (0.653–1.001)	0.041
PLN		< 0.001		0.006		< 0.001		< 0.001
Low	Ref		Ref		Ref		Ref	
Medium	1.685 (0.879–3.265)	0.122	4.768 (1.812–12.548)	0.002	0.915 (0.490–1.711)	0.782	0.952 (0.410–2.209)	0.909
High	0.434 (0.309–0.609)	< 0.001	0.678 (0.325–1.416)	0.302	0.199 (0.145–0.271)	< 0.001	0.282 (0.168–0.474)	< 0.001
LNR (≤ 0.22: >0.22)	1.973 (1.674–2.325)	< 0.001	1.348 (0.664–2.737)	0.408	2.257 (1.946–2.618)	< 0.001	0.881 (0.517–1.501)	0.641
Satellite sites (Absence: Presence)	0.705 (0.599–0.831)	< 0.001	0.774 (0.496–1.209)	0.260	0.466 (0.350–0.621)	< 0.001	0.914 (0.759–1.101)	0.344
Tumor differentiation		0.060				0.015		0.002
Well	Ref				Ref		Ref	
Moderate	1.417 (1.004–1.999)	0.047			1.427 (1.060–1.922)	0.019	1.743 (1.049–2.899)	0.032
Poor	0.492 (0.119–2.030)	0.327			0.373 (0.091–1.528)	0.171	0.598 (0.227–1.575)	0.298
Microvascular invasion (Absence: Presence)	0.789 (0.647–0.963)	0.020	1.008 (0.623–1.630)	0.975	0.755 (0.637–0.895)	0.001	1.037 (0.844–1.274)	0.730
Lymph-vessel invasion (Absence: Presence)	0.823 (0.625–1.084)	0.166			0.898 (0.691–1.169)	0.425		
Macrovascular invasion (Absence: Presence)	0.808 (0.595–1.099)	0.175			0.840 (0.645–1.092)	0.193		
Adjacent organ invasion (Absence: Presence)	0.753 (0.597–0.949)	0.016	0.696 (0.361–1.341)	0.279	0.725 (0.595–0.884)	0.001	0.860 (0.660–1.122)	0.266
Liver capsule invasion (Absence: Presence)	0.898 (0.760–1.061)	0.205		0.134	0.848 (0.734–0.980)	0.025	0.972 (0.616–1.535)	0.904
T stage 8th		0.010		0.258		0.015		0.861
1	Ref		Ref		Ref		Ref	
2	0.246 (0.107–0.567)	0.001	0.514 (0.181–1.459)	0.211	0.395 (0.210–0.745)	0.004	1.346 (0.637–2.844)	0.436
3	0.575 (0.290–1.140)	0.113	1.704 (0.173–16.809)	0.648	0.514 (0.282–0.938)	0.030	0.848 (0.159–4.526)	0.847
4	0.837 (0.444–1.579)	0.583	0.324 (0.105–0.995)	0.049	0.778 (0.447–1.353)	0.374	0.842 (0.406–1.746)	0.644
5	0.717 (0.411–1.250)	0.241	0.778 (0.310–1.952)	0.592	0.799 (0.500-1.277)	0.348	1.094 (0.483–2.474)	0.830
Tumor size (≤ 5 cm: >5 cm)	1.703 (1.413–2.052)	< 0.001	1.160 (0.722–1.863)	0.539	1.471 (1.267–1.708)	< 0.001	1.244 (1.025–1.511)	0.027
Ln 5 (Absence: Presence)	0.998 (0.496–2.007)	0.995			0.862 (0.520–1.429)	0.565		
Ln 6 (Absence: Presence)	0.552 (0.206–1.482)	0.238			0.504 (0.188–1.354)	0.174		
Ln 7 (Absence: Presence)	0.987 (0.491–1.984)	0.970			0.823 (0.496–1.365)	0.451		
Ln 8 (Absence: Presence)	0.775 (0.637–0.944)	0.011	0.504 (0.294–0.863)	0.013	0.595 (0.504–0.702)	< 0.001	0.878 (0.704–1.097)	0.252
Ln 9 (Absence: Presence)	0.561 (0.358–0.880)	0.012	0.588 (0.191–1.809)	0.355	0.660 (0.439–0.992)	0.046	0.800 (0.480–1.334)	0.393
Ln 10 (Absence: Presence)	0.719 (0.269–1.926)	0.512			0.732 (0.274–1.958)	0.534		
Ln 12 (Absence: Presence)	0.692 (0.583–0.821)	< 0.001	1.161 (0.675–1.997)	0.590	0.589 (0.507–0.685)	< 0.001	0.979 (0.769–1.247)	0.864
Ln 13 (Absence: Presence)	0.844 (0.667–1.069)	0.160			0.704 (0.580–0.854)	< 0.001	1.030 (0.808–1.314)	0.809
Ln 14 (Absence: Presence)	4.497 (0.005-4210.085)	0.667			0.556 (0.207–1.491)	0.243		
Ln 16 (Absence: Presence)	0.475 (0.353–0.640)	< 0.001	0.541 (0.226–1.296)	0.169	0.461 (0.352–0.605)	< 0.001	0.821 (0.577–1.170)	0.276
TNM 8th		< 0.001		0.341		< 0.001		0.966
IA	Ref		Ref		Ref		Ref	
IB	0.168 (0.081–0.345)	< 0.001	0.514 (0.181–1.459)	0.211	0.276 (0.161–0.472)			
IIA	0.384 (0.221–0.669)	0.001	0.470 (0.051–4.323)	0.505	0.352 (0.212–0.584)		1.221 (0.150–9.942)	0.852
IIB	0.447 (0.262–0.761)	0.003	2.560 (1.018–6.436)	0.046	0.444 (0.274–0.721)		1.265 (0.609–2.627)	0.529
IIIA	0.000	0.953	0.000	0.965	0.000	0.940	0.000	0.959
IIIB	0.412 (0.277–0.611)	< 0.001	1.039 (0.532–2.031)	0.091	0.485 (0.344–0.686)	< 0.001	0.942 (0.542–1.638)	0.833
After operation therapy (no: yes)	0.725 (0.616–0.854)	< 0.001	1.189 (0.798–1.771)	0.394	0.561 (0.485–0.650)	< 0.001	0.770 (0.646–0.918)	0.004

Abbreviations: OS, overall survival; PFS, progression-free survival; Ref, reference;

### Conduction of nomogram

**Fig. 3:**
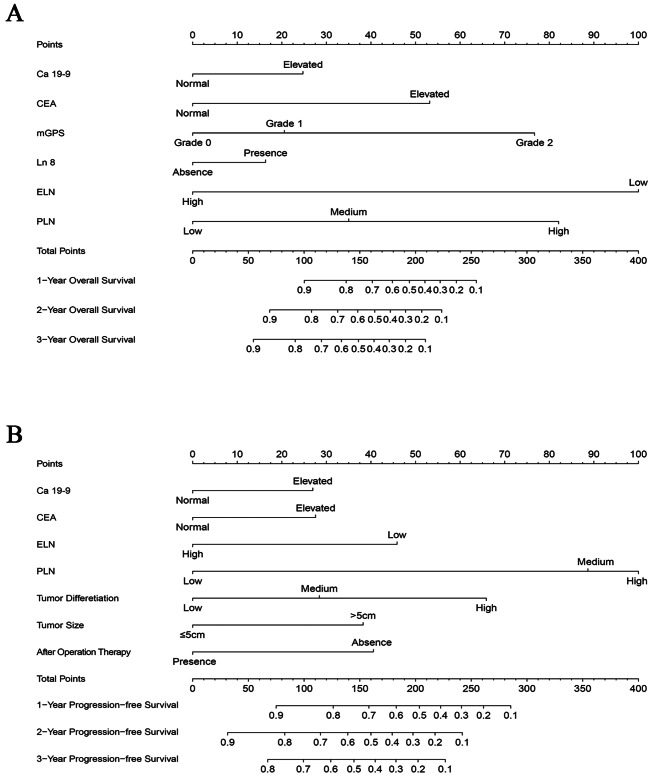
A novel nomogram for predicting the OS (**A**) and PFS (**B**) in iCCA patients based on the modified PLN staging system

Two nomograms were conducted with the independent prognostic factors defined in the Cox-regression analysis to predict 2-, 3-, and 4-year OS and 1-, 2-, 3-year PFS for iCCA patients (Fig. 3). This nomogram could evaluate the probability of survival outcomes by adding up the scores for each variable.

### Validation of the novel nomogram

**Fig. 4:**
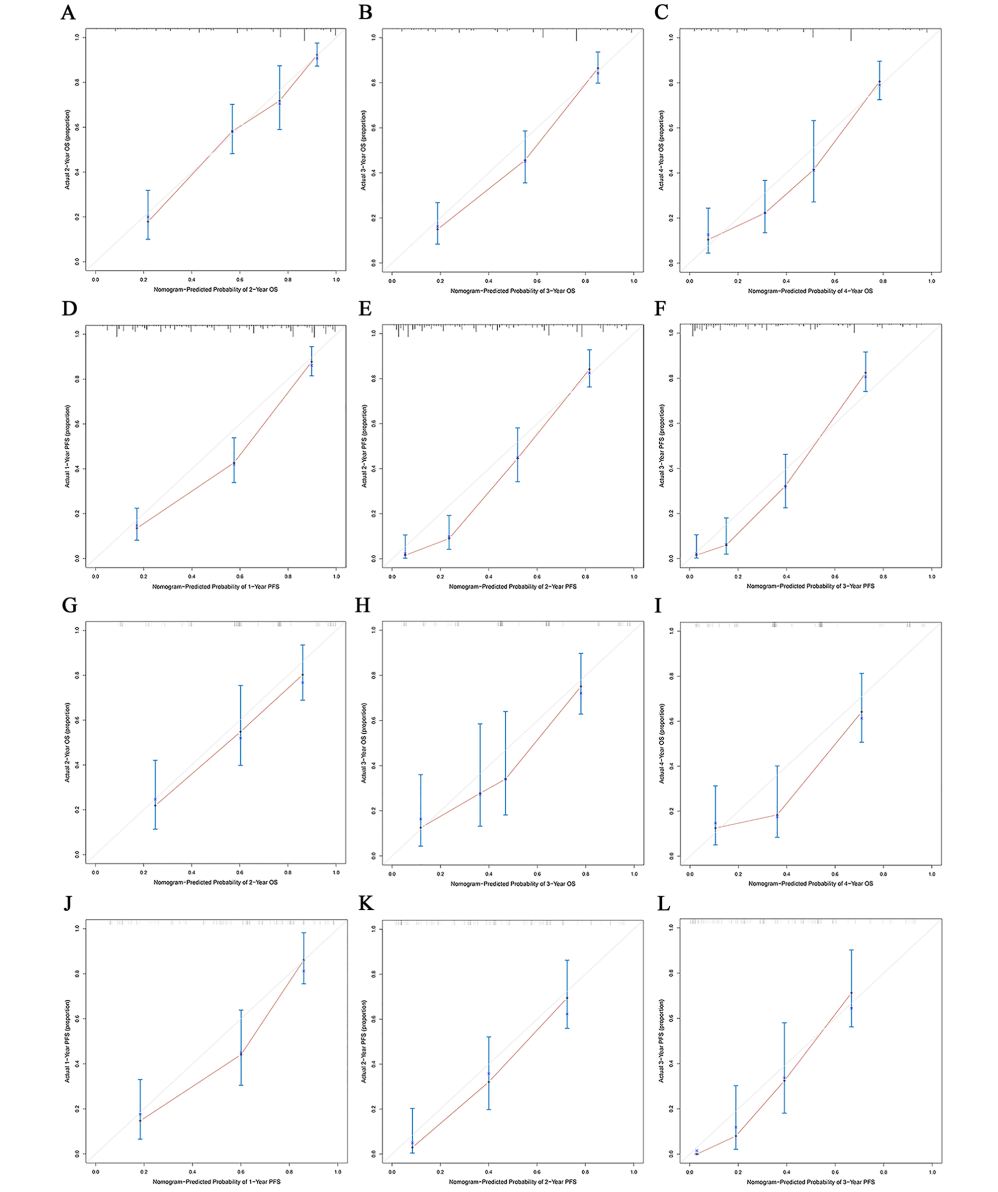
The calibration curve for predicting OS (**A**, **B**, **C**) and PFS (**D**, **E**, **F**) in the training cohort, OS (**G**, **H**, **I**) and PFS (**J**, **K**, **L**) in the validation cohort, respectively

**Fig. 5:**
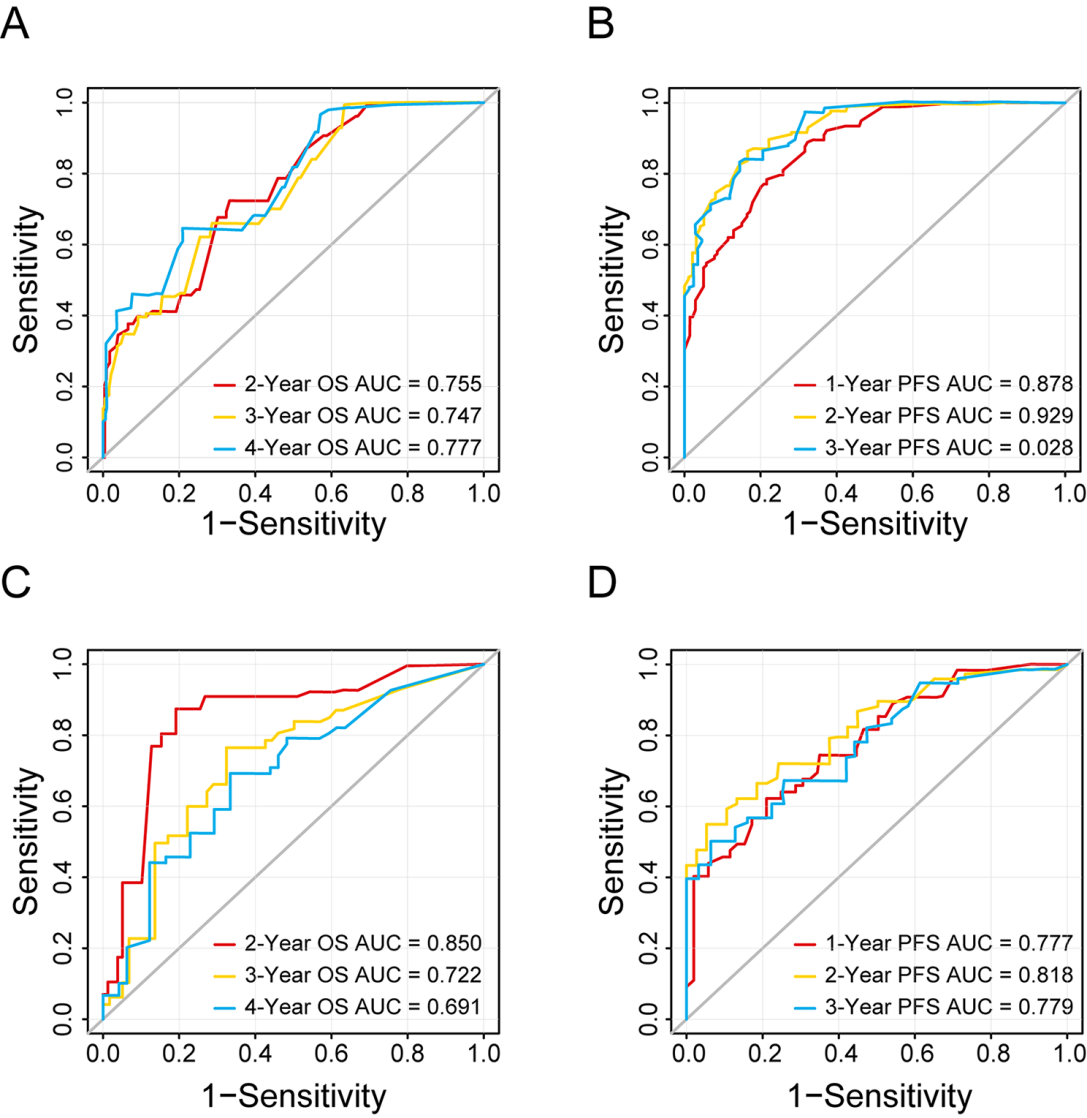
ROC curves of OS (**A**) and PFS (**B**) in the primary cohort. ROC curves of OS (**C**) and PFS (**D**) in the validation cohort

These two nomograms were further validated in our primary and validation cohorts. As shown in Fig. 4, the calibration curves demonstrated an objective agreement between actual and predicted survival of both primary and validation cohorts. In terms of OS prediction, the C-indexes of the nomogram were 0.813 in the primary cohort and 0.787 in the validation cohort. As for PFS prediction, the C-indexes of this novel nomogram were 0.869 in the primary cohort and 0.762 in the validation cohort. ROC curves and AUROC values were also calculated to exhibit the prognostic capacity of the nomogram, as presented in Fig. 5.

## Discussion

Intrahepatic cholangiocarcinoma, as a subtype of hepatobiliary malignancies, owned an increasing incidence worldwide [13]. Those patients with iCCA often suffer from worse overall survival and progression-free survival in contrast with the patients with HCC [14]. Although there were certain similarities in anatomical location between iCCA and HCC, their histological origin and biological behavior were quite varying [9]. Especially for the lymph nodes metastasis, it could be observed in up to 40% of iCCA patients [15–17]. Thus, Ln dissection played a more vital role in iCCA surgery than it did in HCC. And sufficiently Ln dissection did earn a better survival outcome in iCCA patients than insufficiently Ln dissection or non-Ln dissection did [18–20]. As an evaluation of the results of Ln dissection, the present N staging system was quietly different from those in other BTC. According to the 8th TNM staging system, the N stage of extrahepatic bile duct carcinoma and gallbladder carcinoma were both classified into N0 (PLN = 0), N1 (PLN 1–3), and N2 (PLN 4 or more). There were limited studies that classified the N stage of iCCA into 3 groups like other BTC. In the present study, we set the cut-off value of PLN as 3 for the first time. Then the prognostic capacity of the new classification of PLN and the current N stage system of 8th TNM was compared in our iCCA patients. According to the modified classification of PLN, the contrastive analysis of plenty of clinical characteristics was performed in different groups. Finally, a novel nomogram that could accurately stratify patients into subgroups with distinct prognoses based on the new PLN classification was established and validated.

There were similar risk factors between iCCA and HCC, including chronic viral hepatitis, cirrhosis, and alcohol excess [4, 21], this could further indicate the common pathobiological pathways to all primary liver parenchymal malignances. However, different from HCC, which is derived from hepatocytes, iCCA resulted from malignant transformation of cholangiocytes [22]. Whereas two recent studies indicated that iCCA may also arise from trans-differentiation of hepatocytes [2, 3], this needs to be further verified in clinical specimens. Influenced by the different origination, iCCA owned a much higher prevalence of lymph node metastasis than HCC [14].

Given that Ln metastasis is more prevalent in iCCA than in HCC, and the demonstrated powerful prognostic role of Ln metastasis for iCCA, lymphadenectomy was strongly considered at the time of surgery [1]. The fact that metastatic lymph nodes were found in nearly 30% of patients who received lymphadenectomy at the time of surgery could further testify to the necessity of Ln dissection [23]. Moreover, there was a single-center retrospective study obtained that in the sufficient lymphadenectomy group (Dissected Ln > 6) and non-lymphadenectomy group, the former’s OS was much better [20]. This research indicated the importance of standard lymphadenectomy. In contrast to this recommendation of lymph nodes dissection, some researchers argued that the necessity of routine lymphadenectomy in patients with non-clinically apparent lymph node metastasis remains to be proven [24]. However, in a recent study that compared the effect of Ln dissection on survival outcomes in iCCA patients with no suspect Ln metastasis before surgery, the results demonstrated that Ln dissection could improve both overall survival and progression-free survival [18]. In consideration of these recent researches and guidelines, we recommended conducting lymphadenectomy of regional nodes routinely in all iCCA patients.

PLN, as another indicator to evaluate the status of lymph nodes, was also identified as a predictive factor for the survival of patients with other types of malignancies. The present lymph nodes staging system with a cut-off value of 1 (i.e. Positive or Negative) had been proven to stratify the patients’ survival outcomes effectively in several studies [25], and this result was also verified in our research. Nevertheless, given the more lymphatic involvement of iCCA and the powerful prognostic capacity of PLN, the staging system’s stratifying ability of PLN in iCCA should be improved. Some researchers attempted to set a novel valid cut-off value of PLN [16, 26]. However, in these limited studies, the cut-off values of PLN varied. Of note, most of these studies were single-center studies with limited cohorts. To our latest knowledge, our study was the first to stratify the PLN classification into 3 groups with a cut-off value of 3 and further conduct a nomogram to predict its impact on survival outcomes of iCCA patients based on multi-center cohorts.

There were several limitations in the present study. First, although this study was conducted based on large cohorts of multi-center, all patients were from China. The larger cohorts from different countries and regions were required to further verify the prognostic capacity of this nomogram. Second, on account of the long-time duration of this study, not all the patients received the standard Ln dissection in surgery, there may be bias in the analysis of ELN. A detailed record with standard lymphadenectomy is warranted to obtain additional objective information. Third, the retrospective data of surgery, especially the location of dissected lymph nodes, sometimes can be obscure. The lack of detailed information makes it difficult to analyze the location of PLN’s impact toal survival outcomes. Last, the systematic bias of the retrospective study caused by the incomplete adherence to follow-up protocol also exists in this present study.

## Conclusion

The modified classification of PLN in iCCA could further stratify the patients with the N1 stage in 8th TNM staging system into two groups with different survival outcomes. Moreover, the different grades of the new classification of PLN correlated with different levels of adverse clinical characteristics remarkably. Based on the new classification of PLN in iCCA, a novel nomogram was conducted to predict survival outcomes in iCCA patients. It exhibited remarkably accuracy of prognostic prediction. Accordingly, frequent monitoring should be taken in the patients with the higher score in this nomogram.

## Electronic supplementary material

Below is the link to the electronic supplementary material.

Supplementary Material 1

## Data Availability

The datasets generated and/or analyzed during the current study are not publicly available but are available from the corresponding author on reasonable request.

## References

[1] Bridgewater J, Galle PR, Khan SA, Llovet JM, Park JW, Patel T, Pawlik TM, Gores GJ (2014). Guidelines for the diagnosis and management of intrahepatic cholangiocarcinoma. J Hepatol.

[2] Fan B, Malato Y, Calvisi DF, Naqvi S, Razumilava N, Ribback S, Gores GJ, Dombrowski F, Evert M, Chen X, Willenbring H (2012). Cholangiocarcinomas can originate from hepatocytes in mice. J Clin Invest.

[3] Sekiya S, Suzuki A (2012). Intrahepatic cholangiocarcinoma can arise from Notch-mediated conversion of hepatocytes. J Clin Invest.

[4] Khan SA, Thomas HC, Davidson BR, Taylor-Robinson SD (2005). Cholangiocarcinoma The Lancet.

[5] Khan SA, Toledano MB, Taylor-Robinson SD (2008). Epidemiology, risk factors, and pathogenesis of cholangiocarcinoma. HPB (Oxford).

[6] Yasser Shaib HBE-S. The Epidemiology of Cholangiocarcinoma, SEMINARS IN LIVER DISEASE, 24 (2004). 10.1055/s-2004-828889 15192785

[7] Lee AJ, Chun YS (2018). Intrahepatic cholangiocarcinoma: the AJCC/UICC 8th edition updates. Chin Clin Oncol.

[8] Chun YS, Pawlik TM, Vauthey JN (2018). 8th Edition of the AJCC Cancer staging Manual: pancreas and hepatobiliary cancers. Ann Surg Oncol.

[9] Mejia JC, Pasko J (2020). Primary liver cancers: Intrahepatic Cholangiocarcinoma and Hepatocellular Carcinoma. Surg Clin North Am.

[10] Gao P, Zhu T, Gao J, Li H, Liu X, Zhang X (2021). Impact of examined Lymph Node Count and Lymph Node Density on overall survival of Penile Cancer. Front Oncol.

[11] He C, Mao Y, Wang J, Huang X, Lin X, Li S (2018). Surgical management of periampullary adenocarcinoma: defining an optimal prognostic lymph node stratification schema. J Cancer.

[12] Li J, Sun Y, Zhao B, Tang C, Fan D, Jiang W, Rixiati Y (2020). Lymph node ratio-based staging system for Gallbladder Cancer with fewer than six lymph nodes examined. Front Oncol.

[13] Endo I, Gonen M, Yopp AC, Dalal KM, Zhou Q, Klimstra D, D’Angelica M, DeMatteo RP, Fong Y, Schwartz L, Kemeny N, O’Reilly E, Abou-Alfa GK, Shimada H, Blumgart LH, Jarnagin WR (2008). Intrahepatic cholangiocarcinoma: rising frequency, improved survival, and determinants of outcome after resection. Ann Surg.

[14] Zhou XD, Tang ZY, Fan J, Zhou J, Wu ZQ, Qin LX, Ma ZC, Sun HC, Qiu SJ, Yu Y, Ren N, Ye QH, Wang L, Ye SL (2009). Intrahepatic cholangiocarcinoma: report of 272 patients compared with 5,829 patients with hepatocellular carcinoma. J Cancer Res Clin Oncol.

[15] Wang Y, Li J, Xia Y, Gong R, Wang K, Yan Z, Wan X, Liu G, Wu D, Shi L, Lau W, Wu M, Shen F (2013). Prognostic nomogram for intrahepatic cholangiocarcinoma after partial hepatectomy. J Clin Oncol.

[16] Hu H, Xu G, Du S, Luo Z, Zhao H, Cai J (2021). The role of lymph node dissection in intrahepatic cholangiocarcinoma: a multicenter retrospective study. BMC Surg.

[17] Navarro JG, Lee JH, Kang I, Rho SY, Choi GH, Han DH, Kim KS, Choi JS. Prognostic significance of and risk prediction model for lymph node metastasis in resectable intrahepatic cholangiocarcinoma: do all require lymph node dissection?, HPB (Oxford), 22 (2020) 1411–9. 10.1016/j.hpb.2020.01.009 32046923

[18] Yoh T, Cauchy F, Le Roy B, Seo S, Taura K, Hobeika C, Dokmak S, Farges O, Gelli M, Sa Cunha A, Adam R, Uemoto S, Soubrane O (2019). Prognostic value of lymphadenectomy for long-term outcomes in node-negative intrahepatic cholangiocarcinoma: a multicenter study. Surgery.

[19] Ma WJ, Wu ZR, Hu HJ, Wang JK, Yin CH, Shi YJ, Li FY, Cheng NS (2020). Extended Lymphadenectomy Versus Regional Lymphadenectomy in Resectable Hilar Cholangiocarcinoma. J Gastrointest Surg.

[20] Kim SH, Han DH, Choi GH, Choi JS, Kim KS (2019). Oncologic impact of Lymph Node Dissection for Intrahepatic Cholangiocarcinoma: a Propensity score-matched study. J Gastrointest Surg.

[21] Palmer WC, Patel T (2012). Are common factors involved in the pathogenesis of primary liver cancers? A meta-analysis of risk factors for intrahepatic cholangiocarcinoma. J Hepatol.

[22] Blechacz B, Komuta M, Roskams T, Gores GJ (2011). Clinical diagnosis and staging of cholangiocarcinoma. Nat Rev Gastroenterol Hepatol.

[23] Nathan H, Aloia TA, Vauthey JN, Abdalla EK, Zhu AX, Schulick RD, Choti MA, Pawlik TM (2009). A proposed staging system for intrahepatic cholangiocarcinoma. Ann Surg Oncol.

[24] de Jong MC, Nathan H, Sotiropoulos GC, Paul A, Alexandrescu S, Marques H, Pulitano C, Barroso E, Clary BM, Aldrighetti L, Ferrone CR, Zhu AX, Bauer TW, Walters DM, Gamblin TC, Nguyen KT, Turley R, Popescu I, Hubert C, Meyer S, Schulick RD, Choti MA, Gigot JF, Mentha G, Pawlik TM (2011). Intrahepatic cholangiocarcinoma: an international multi-institutional analysis of prognostic factors and lymph node assessment. J Clin Oncol.

[25] Spolverato G, Kim Y, Alexandrescu S, Marques HP, Lamelas J, Aldrighetti L, Clark Gamblin T, Maithel SK, Pulitano C, Bauer TW, Shen F, Poultsides GA, Tran TB, Wallis Marsh J, Pawlik TM (2016). Management and outcomes of patients with recurrent Intrahepatic Cholangiocarcinoma following previous curative-intent Surgical Resection. Ann Surg Oncol.

[26] Kim SH, Han DH, Choi GH, Choi JS, Kim KS (2022). Extent of Lymph Node Dissection for Accurate Staging in Intrahepatic Cholangiocarcinoma. J Gastrointest Surg.

