# Supplementary figures and images for: Modified staging system of positive lymph nodes based nomogram in intrahepatic cholangiocarcinoma
Source: Cancer Cell Int. 2023 Jul 29;23:148. doi: 10.1186/s12935-023-03005-6 (PMC10386250; doi:10.1186/s12935-023-03005-6)

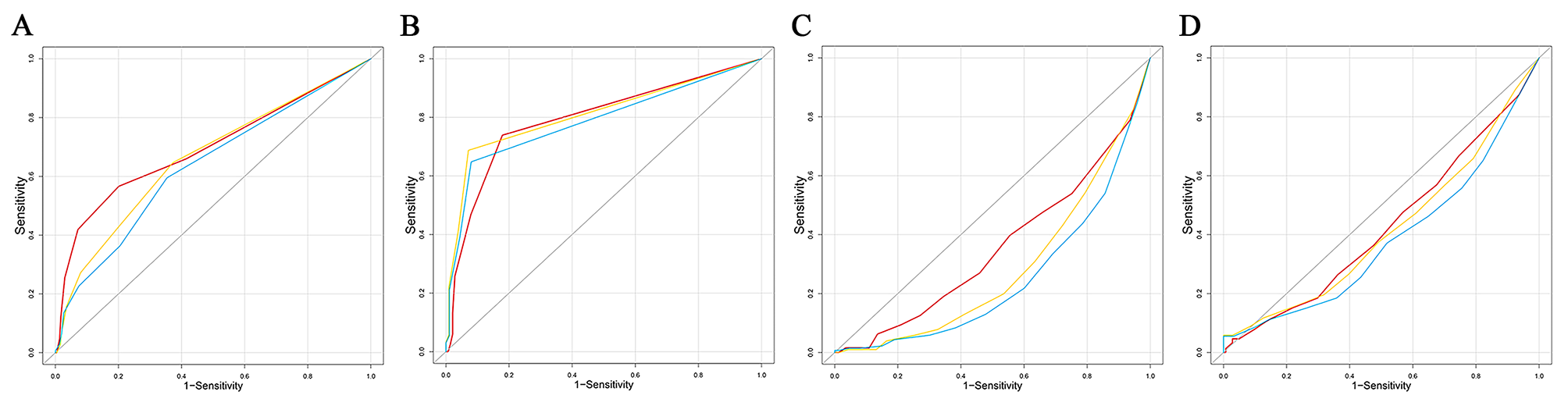

Supplement: Supplementary file 1 — Supplementary Material 1 [file 12935_2023_3005_MOESM1_ESM.png]
